# Protein Cage-like Vesicles Fabricated via Polymerization-Induced Microphase Separation of Amphiphilic Diblock Copolymers

**DOI:** 10.3390/ma18030727

**Published:** 2025-02-06

**Authors:** Eri Yoshida

**Affiliations:** Department of Applied Chemistry and Life Science, Toyohashi University of Technology, 1-1 Hibarigaoka, Tempaku-cho, Toyohashi 441-8580, Japan; yoshida.eri.gu@tut.jp

**Keywords:** artificial protein models, protein cage, clathrin-like morphology, polymer vesicles, amhiphilic diblock copolymer, porous vesicles, microphase separation, triskelion-like segments, polymerization-induced microphase separation, polymerization-induced self-assembly

## Abstract

Highly symmetric protein cages represent one of the most artistic architectures formed by biomolecules. However, the underlying reasons for the formation of some of these architectures remain unknown. The present study aims to investigate the significance behind their morphological formation by fabricating protein cage-like vesicles using a synthetic polymer. The vesicles were synthesized by combining polymerization-induced self-assembly (PISA) with polymerization-induced microphase separation (PIMS), employing an amphiphilic poly(methacrylic acid)-*block*-poly(*n*-butyl methacrylate-*random*-cyclohexyl methacrylate-*random*-methacrylic acid) diblock copolymer, PMAA-*b*-P(BMA-*r*-CMA-*r*-MAA). The copolymer, with a 60 mol% molar ratio of CMA to the BMA units, produced clathrin-like vesicles with angular windows in their shell, resulting from the segregation of the hard CMA units from the soft BMA matrix in the hydrophobic phase of the vesicle. These vesicles were highly stable against rising temperatures. In contrast, the vesicles with a 30 mol% CMA ratio dissociated upon heating to 50 °C into triskelion-like segments due to intramolecular microphase separation. These findings indicate that designing synthetic polymers can mimic living organ morphologies, aiding in elucidating their morphological significance and inspiring the development of new materials utilizing these morphologies.

## 1. Introduction

Protein cages are hollow containers formed by the self-assembly of single or multiple proteins. These highly symmetrical and monodispersed cages, with regular holes on their shells, exhibit various morphologies such as icosahedrons, spheres, and rods, depending on the shape and assembly manner of their constituent protein subunits [1,2,3,4]. The cages incorporate substances essential for life activities within their hollow space, storing them in isolation from the outside environment and delivering them to necessary locations. For instance, ferritins, spherical cages composed of 24 protein subunits, store iron ions within their inner space, while simultaneously water-solubilizing and detoxifying the ions and releasing them as needed [5,6]. The small heat-shock protein with chaperone activity from *M. jannaschii* is also a spherical cage composed of 24 subunits and features 8 triangular and 6 square holes on its surface [7]. Viral capsids, such as those of the brome mosaic virus [8] and cowpea chlorotic mottle virus [9], are icosahedral, while the capsid of the tobacco mosaic virus is rod-like [10,11]. These plant viruses encode viral genes within their capsids to facilitate reproduction in host cells. Furthermore, clathrins, composed of 36 protein complexes with a triskelion structure, form dynamic protein cages. These temporarily assemble to enhance endocytosis by coating transport vesicles and subsequently dissociate into triskelion subunits after delivering the vesicle to a target organelle or the plasma membrane [12,13,14,15].

Mimicking the protein cages in morphology and formation mechanisms has led to the creation of porous microparticles as well as artificial protein cages [16,17,18,19] for biomedical and therapeutic applications [20]. The preparation methods for porous particles using bio-based oligomers or synthetic polymers are mainly classified into two categories: the double emulsion-solvent evaporation technique [21,22,23] and emulsion polymerization for PIMS followed by etching [24]. While the former technique can utilize bio-based polymers, such as polypeptides prepared by polycondensation, the latter offers advantages in designing a wide range of synthetic block copolymers through the controlled/living radical polymerization of numerous vinyl monomers [25,26,27,28]. The PIMS technique is useful not only for porous material preparation but also for creating nanoarchitectures of great variety based on microphase separation [29,30,31], including highly conductive ion transport membranes [32,33].

Polymer vesicles composed of amphiphilic block copolymers have significant applications as carriers of functional molecules, such as drugs, genes, and biomarkers, in medical and industrial fields due to their capacity to load both hydrophobic and hydrophilic substances, their mechanical stability, readily controllable dissociation, and broad designability through the tailored design of constituent copolymers [34]. Particularly, micron-sized giant polymer vesicles prepared through PISA effectively serve as artificial models of biomembranes for cells and organelles due to their similarities in size, structure, and stimuli-responsiveness [35,36,37]. The giant vesicles formed morphologies of static and dynamic biomembranes, including villus-like structures [38], erythrocyte-like morphology transformations [37], Golgi apparatus- and endoplasmic reticulum-like anastomosed tubular networks continuous with fenestrated sheets [39], and nuclear membrane-like spherical vesicles with numerous pores on their shells [40]. 

As multiform biomembrane morphologies are based on amphiphilic lipids, diverse high-dimensional structures formed by proteins, such as folding and subunit formation, are attributed to the amphiphilicity of proteins. To understand why the protein forms such a morphology, the present study demonstrates the fabrication of a clathrin-like cage using an amphiphilic block copolymer. The fabrication combines PISA and PIMS for the simultaneous progress of self-assembly of the hydrophobic block with microphase separation in the hydrophobic phase of the resulting vesicle, as the proteins form their cages through one-step self-assembly. Extending the artificial biomembrane model to protein models is helpful in elucidating the intrinsic role of morphology. This paper describes the fabrication of a clathrin-like cage morphology employing an amphiphilic PMAA-*b*-P(BMA-*r*-CMA-*r*-MAA) diblock copolymer through the microphase separation of the hard CMA units from the soft BMA unit matrix in the hydrophobic block phase of the vesicle. 

## 2. Materials and Methods

### 2.1. Vesicle Preparation: General Procedure

Monomers (MAA, BMA, and CMA) and solvents (methanol (MeOH) and water) were purified using conventional methods (see Supplementary Materials). Vesicles were prepared by PISA using PMAA end-capped with 4-methoxy-2,2,6,6-tetramethylpiperidine-1-oxyl (MTEMPO), denoted as PMAA-MTEMPO, through nitroxide-mediated photo-controlled/living radical polymerization (photo-NMP). The PMAA was prepared as reported previously [41]. 2,2′-Azobis[2-(2-imidazolin-2-yl)propane] (Wako Pure Chemical Industries, V-61; 22.8 mg, 0.0911 mmol), MTEMPO [42] (18.0 mg, 0.0966 mmol), (4-*tert*-butylphenyl)diphenylsulfonium triflate (Sigma-Aldrich, *^t^*BuS; 24.0 mg, 0.0512 mmol), MAA (Wako Pure Chemical Industries; 2.030 g, 23.6 mmol), and MeOH (4 mL) were placed in a 30 mL test tube connected to a high-vacuum valve. The contents were degassed several times using a freeze–pump–thaw cycle and then charged with nitrogen. The polymerization was carried out at room temperature for 5.5 h with irradiation at 9.0 amperes by reflective light from a mirror using a BA-H502 Ushio optical modulex, an OPM2-502H illuminator with a UI-OP2SL high-illumination lens, and a 500 W super high-pressure UV lamp (USH-500SC2, Ushio Co., Ltd.) to avoid any side reactions caused by direct irradiation [43]. MeOH (11 mL) and water (5 mL), degassed by bubbling argon for 15 min, were added to the product under a flow of argon. After the product was completely dissolved in the aqueous MeOH solution, part of the mixture (ca. 1 mL) was withdrawn to determine the monomer conversion and molecular weight (*M*_n_) of the resulting PMAA-MTEMPO. The solution withdrawn was poured into ether (50 mL) to precipitate the polymer. The precipitate was collected by filtration and dried in vacuo for several hours to obtain the PMAA (103.8 mg). The monomer conversion (79%) was determined by ^1^H NMR using a Jeol ECS500 FT NMR spectrometer, while the molecular weight (*M*_n_ = 19,050) and its polydispersity index (*M*_w_/*M*_n_ = 1.57) were estimated by gel permeation chromatography (GPC) based on PMAA standards using a Tosoh GPC-8020 instrument equipped with a DP-8020 dual pump, a CO-8020 column oven, and an RI-8020 refractometer. Two gel columns, Tosoh TSK-GEL α-M, were used with *N*,*N*-dimethylformamide containing 30 mM LiBr and 60 mM H₃PO₄ as the eluent at 40°C. The degree of polymerization (DP = 218) for the PMAA was calculated based on the molecular weights of the PMAA, the initiator fragment, and MTEMPO. The concentration of the PMAA was estimated to be 4.83 mM, based on the initial concentration of MTEMPO [44], disregarding a slight contraction in volume due to the mixing of MeOH and water (Supplementary Materials).

The PMAA solution (4 mL, containing 1.93 × 10^−2^ mmol of PMAA and 0.988 mmol of unreacted MAA, based on a monomer conversion of 79%), BMA (Wako Pure Chemical Industries; 205.6 mg, 1.446 mmol), CMA (Wako Pure Chemical Industries; 366.3 mg, 2.177 mmol), and MAA (314.7 mg, 3.655 mmol) were placed in a 30 mL test tube connected to a high-vacuum valve under a flow of argon. The contents were degassed several times using a freeze–pump–thaw cycle and then charged with nitrogen. The block copolymerization was carried out for 8 h at room temperature and 600 rpm by irradiation at 9.0 amperes using reflective light from a mirror with a high-pressure mercury lamp. After the polymerization, part of the resulting dispersion solution (ca. 0.3 mL) was withdrawn to determine the monomer conversions (BMA: 78%, CMA: 84%, and MAA: 40%). A mixed solvent (MeOH/H₂O = 3/1 *v*/*v*, 20 mL) was added to the dispersion solution to precipitate vesicles. The vesicles were cleaned with the mixed solvent through a repeated sedimentation–redispersion process. The resulting vesicles were stored in the presence of a small amount of the mixed solvent. The molar ratio of units in the random copolymer block was calculated based on the monomer conversions (BMA/CMA/MAA = 0.233/0.377/0.390).

### 2.2. Thermoresponsive Behavior of the Vesicles 

The vesicles (0.8 mg), dried in air, and a 70% aqueous MeOH solution (MeOH/H₂O = 3/1 *v*/*v*, 3 mL) were placed in a 4 mL square glass bottle with a screw cap and left at room temperature for 1 h to disperse the vesicles in the solution. The dispersion of the vesicles was then heated to the designated temperature, maintained at that temperature for 15 min, and subjected to light scattering measurement (nanoSAQLA, Photal Otsuka Electronics, Japan).

### 2.3. Sample Characterization

Differential scanning calorimetry (DSC) was performed on a sample (1.7 mg) at a heating rate of 20 °C/min using Shimadzu DSC-60 instrument equipped with a TA-60WS system controller and an FC-60 nitrogen flow controller. Field emission scanning electron microscopy (FE-SEM) measurements were performed using a Hitachi SU8000 scanning electron microscope at 0.7 kV without coating.

## 3. Results

**Figure 1:**
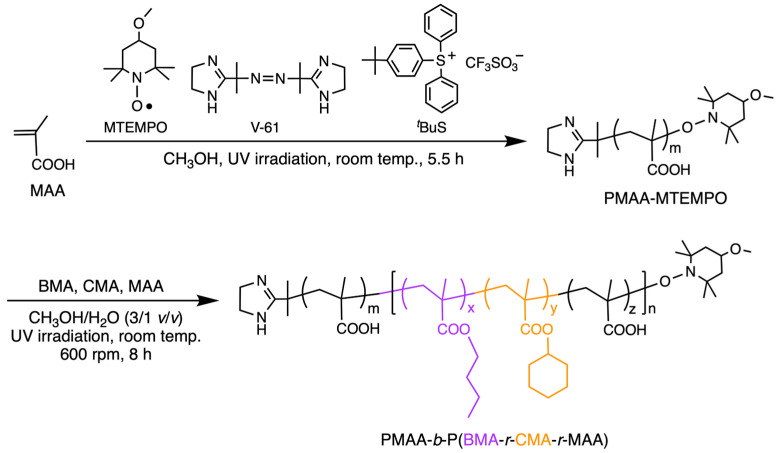
Schematic procedure for the synthesis of PMAA-*b*-P(BMA-*r*-CMA-*r*-MAA) diblock copolymers.

To produce the clathrin cage-like morphology on a vesicle surface through the microphase separation of soft and hard segments in the hydrophobic phase of a vesicle, BMA and CMA were chosen as the components of the hydrophobic block. These soft and hard components have highly hydrophobic side groups, with free energy values (*μ*°_HC_ − *μ*°_W_) of −5972 cal/mol for *n*-butyl and −6730 cal/mol for cyclohexyl [45,46]. However, their homopolymers differ significantly in hardness: a BMA homopolymer has a glass transition temperature (*T*_g_) of 20 °C, while a CMA homopolymer has a *T*_g_ of 83 °C [47,48]. The vesicles, composed of the amphiphilic PMAA-*b*-P(BMA-*r*-CMA-*r*-MAA) diblock copolymer, were synthesized through PISA using photo-NMP (Figure 1). 

**Figure 2:**
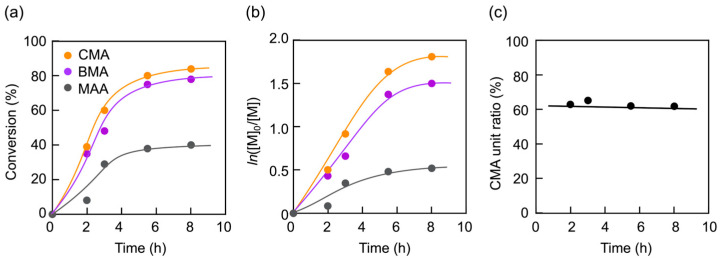
The photo-NMP process for the PISA of PMAA-*b*-P(BMA-*r*-CMA-*r*-MAA): (**a**) time–conversion plots, (**b**) first-order time–conversion plots, and (**c**) variation in the molar ratio of the CMA unit to the total hydrophobic units (BMA and CMA) in the random copolymer block over time.

The block copolymerization was performed using a monomer mixture of BMA, CMA, and MAA at a molar ratio of BMA/CMA/MAA = 0.175/0.263/0.562, with PMAA-MTEMPO as the macroinitiator. The results are summarized in Table S1 (Supplementary Materials). The time–conversion and its first-order plots for the block copolymerization are shown in Figure 2. The MAA conversion reached a steady state at a low level due to increased hydrophobicity in the internal phase, which made it difficult for the hydrophilic MAA to penetrate. In contrast, the conversions of hydrophobic BMA and CMA increased to high levels. The unit proportion of CMA to BMA in the random copolymer block remained constant throughout the polymerization. 

**Figure 3:**
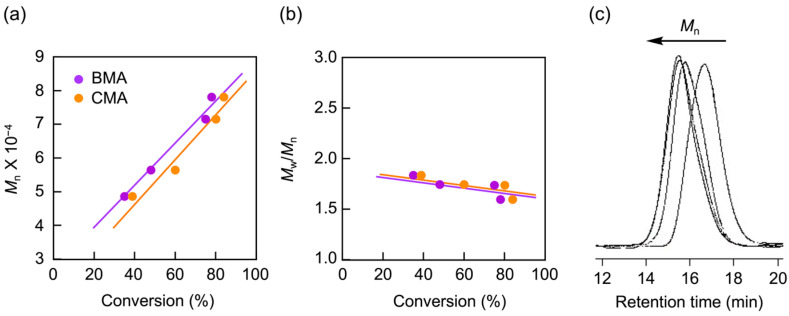
The living nature of the photo-NMP during the PISA of PMAA-*b*-P(BMA-*r*-CMA-*r*-MAA): (**a**) molecular weight plots of the resulting copolymers versus monomer conversions, (**b**) polydispersity index plots of the copolymers versus monomer conversions, and (**c**) GPC profiles of the copolymers after 2, 3, 5.5, and 8 h of polymerization (from right to left).

As shown in Figure 3, the molecular weight of the resulting copolymer linearly increases with the conversions of both BMA and CMA, indicating that the polymerization proceeds via a living mechanism. The polydispersity index (*M*_w_/*M*_n_) slightly decreased as the polymerization progressed, further supporting the living nature of the polymerization. GPC analysis also confirmed the living nature, as the curve of the copolymer shifted toward a higher molecular weight with the monomer conversions. 

**Figure 4:**
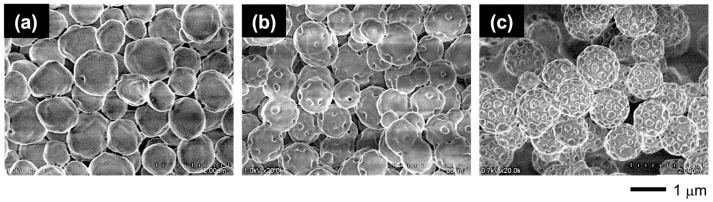
FE-SEM images of the vesicle morphologies at different polymerization times: (**a**) 2 h, (**b**) 3 h, and (**c**) 8 h.

Through this PISA process, the PMAA-*b*-P(BMA-*r*-CMA-*r*-MAA) diblock copolymer produced spherical vesicles. Figure 4 shows variations in the morphology of the vesicles as the polymerization progresses. During the initial stage of polymerization, the vesicles exhibited soft shells with smooth surfaces. As the hydrophobic block chains grew during the polymerization, the vesicles formed several holes on their shells. Further hole formation during the later stages produced porous vesicles with angular windows on their shells.

**Figure 5:**
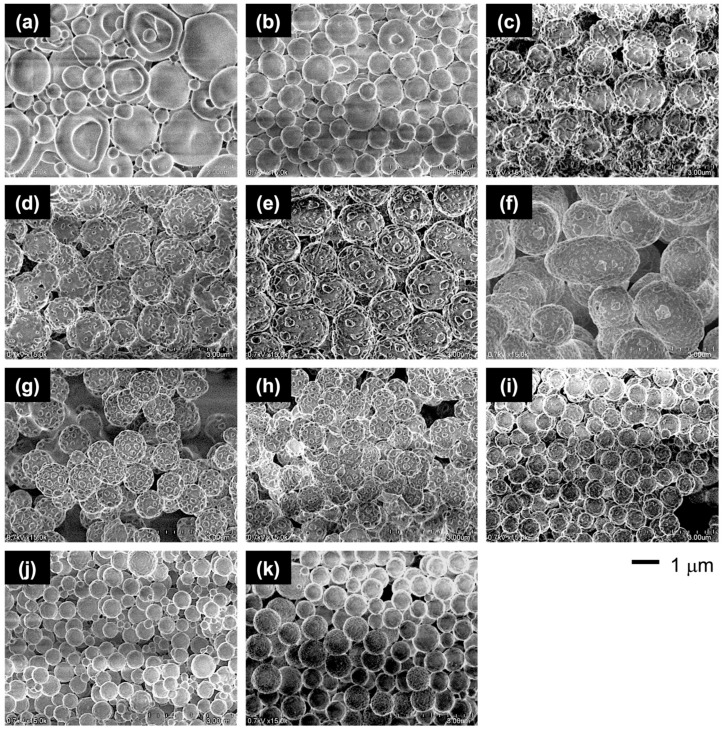
FE-SEM images of the vesicles with varying CMA ratios: (**a**) 0 mol%, (**b**) 10 mol%, (**c**) 20 mol%, (**d**) 30 mol%, (**e**) 40 mol%, (**f**) 50 mol%, (**g**) 60 mol%, (**h**) 70 mol%, (**i**) 80 mol%, (**j**) 90 mol%, and (**k**) 100 mol%.

**Figure 6:**
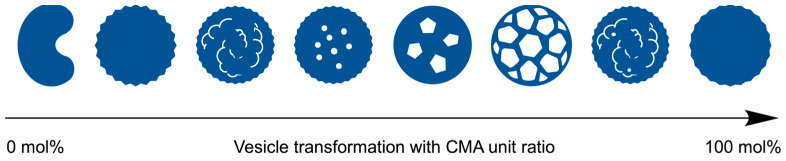
CMA ratio-dependent morphological transformations of the vesicles.

To evaluate the effect of the CMA units on the morphology, the vesicles with different CMA ratios were prepared. The characterizations of the copolymers are summarized in Table 1 and Table S2 (Supplementary Materials). The copolymers had similar chain lengths of the blocks and a similar molar ratio of MAA units in the hydrophobic block, but they differed in their BMA/CMA ratios. The copolymers exhibited different morphologies depending on the CMA ratio (Figure 5). The absence of CMA units resulted in somewhat contorted vesicles with a large dent due to the soft BMA units. Incorporating 10 mol% of CMA units into the hydrophobic block reduced the shell flexibility, making the vesicles more spherical. Increasing the CMA ratio to 30 mol% transformed the smooth shell into a rough surface with small holes. With a further increase to 40 mol%, the small holes grew larger, and at 60 mol%, porous vesicles with many angular windows were produced. The vesicles reverted to rough-surfaced vesicles with small holes at an excessive ratio above 70 mol%. Finally, at 90 mol%, the vesicles lost their holes while maintaining a slightly rough surface (Figure 6).

**Figure 7:**
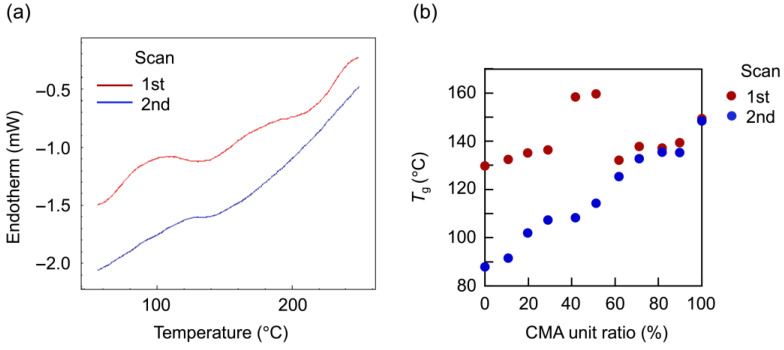
DSC analysis of the vesicles: (**a**) spectra of the vesicles with 60 mol% CMA units, and (**b**) *T*_g_ plots of the hydrophobic blocks from the first and second scans versus the CMA ratio.

**Figure 8:**
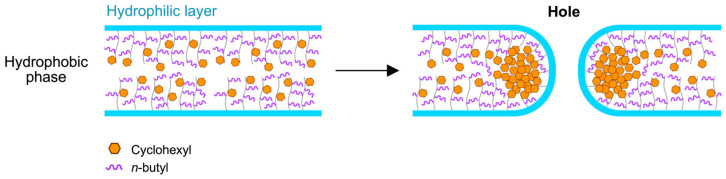
A schematic illustration of hole formation through microphase separation caused by the aggregation of CMA units.

DSC analysis revealed that the hole formation originated from the microphase separation of the hard CMA units in the hydrophobic phase. Figure 7 shows DSC spectra of the vesicles with 60 mol% CMA units. The vesicles exhibited two distinct *T*_g_s originating from the PMAA block and the P(BMA-*r*-CMA-*r*-MAA) block during the first scan. By the second scan, only the *T*_g_ of the random copolymer block was discerned. The *T*_g_s of the random copolymer block from the first and second scans were plotted against the CMA ratio, revealing a large difference in *T*_g_ between the scans at less than 60 mol% CMA. This indicates the presence of well-organized copolymer blocks in the hydrophobic phase of the vesicle. During the first scan, the copolymer blocks were disorganized by melting, resulting in the exhibition of their inherent *T*_g_ based on the molar ratios of monomer units during the second scan. The second *T*_g_ increased with an increasing molar ratio of CMA units. At a 40 mol% CMA ratio, the vesicles exhibited a very high *T*_g_ during the first scan. The copolymer blocks were highly organized in the hydrophobic phase, correlating to the lowest CMA ratio that formed windows in the vesicle shell. The window formation was attributed to deviations in the hydrophobic phase due to the increased CMA units. The hard CMA units aggregated, separating from the copolymer block matrix, causing microphase separation, which led to the formation of windows (Figure 8). The 50 mol% sample also showed a very high *T*_g_ during the first scan due to microphase separation. However, at 60 and 70 mol% CMA ratios, the vesicles did not exhibit very high *T*_g_s during the first scan despite also having windows in their shell. They produced only slight or negligible differences in *T*_g_ between the first and second scans due to the further increase in CMA units. The hard CMA units retained the microphase separation, enhancing the window formation. However, an excessively high CMA ratio reduced deviations in the hydrophobic phase, resulting in the loss of windows.

**Figure 9:**
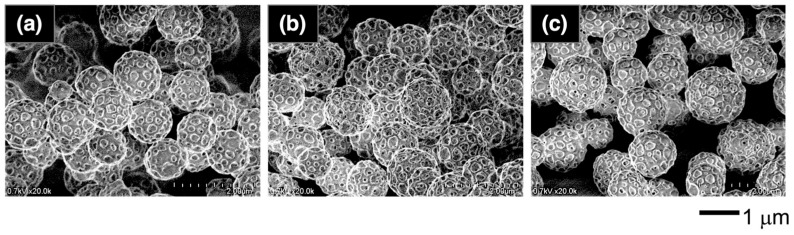
Thermal stability of the vesicles under different conditions: (**a**) 8 g/L, 25 °C; (**b**) 8 g/L, 50 °C for 1 h; and (**c**) 2 g/L, 50 °C for 1 h. CMA units = 60 mol%.

The FE-SEM observations demonstrated that the vesicles with angular windows in their shells were highly stable against rising temperatures. A previous study showed that the vesicles without CMA units transformed into cup-shaped or echinocyte-like vesicles, releasing microspherules when heated in aqueous methanol at 50 °C [37]. However, the vesicles with 60 mol% CMA units exhibited no changes in morphology upon heating (Figure 9). Even diluting the vesicle concentration caused no disruption. 

**Figure 10:**
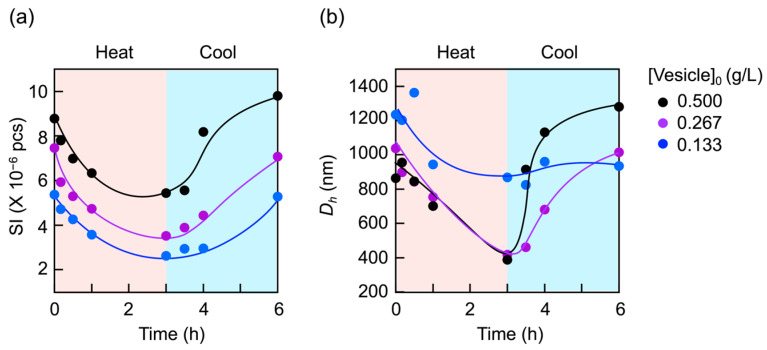
Variations in (**a**) scattering intensity (*SI*) and (**b**) hydrodynamic size (*D*_h_) of the vesicles at different concentrations. CMA units = 30 mol%.

In contrast, the vesicles with 30 mol% CMA units dissociated upon heating. A light scattering study revealed the reversibility of this dissociation. Variations in scattering intensity and hydrodynamic size over time for the 30 mol% sample are shown in Figure 10 when heated at 50 °C, followed by cooling to 25 °C. The intensity and size continued to decrease with time during the heating but increased with time during the cooling, returning to their original values, independent of the vesicle concentration. However, the vesicles returned more quickly at higher concentrations. This dissociation–reassociation was completely reversible and repeatable by alternating the heating and cooling, as evidenced by the clear hysteresis in scattering intensity and size changes during the repeated cycles (Figure S1 in Supplementary Materials).

**Figure 11:**
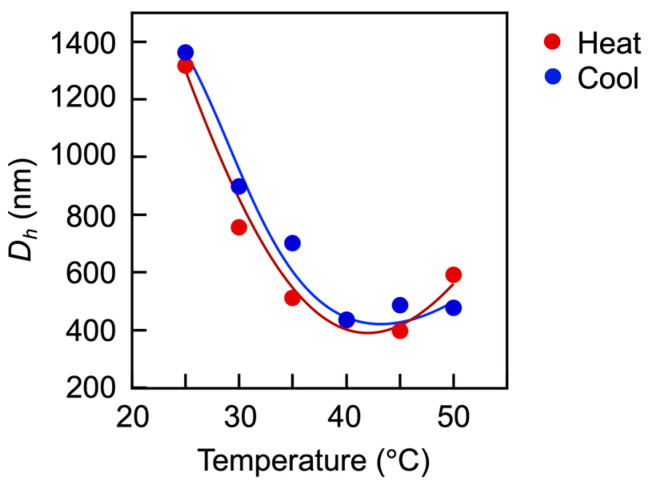
Variation in the *D*_h_ of the vesicles during heating, followed by cooling. The vesicles are maintained at each temperature for 15 min. CMA units = 30 mol%, [vesicle]_0_ = 0.267 g/L.

Detailed investigations of the vesicle disruption clarified the dissociation process. Figure 11 shows plots of the size at each temperature during stepwise heating and cooling. Upon heating, the vesicle size decreased with the temperature rising; however, it slightly increased above 45 °C, suggesting the reassociation of the copolymers. On cooling, the size reverted to its original value, following almost the same course. 

**Figure 12:**
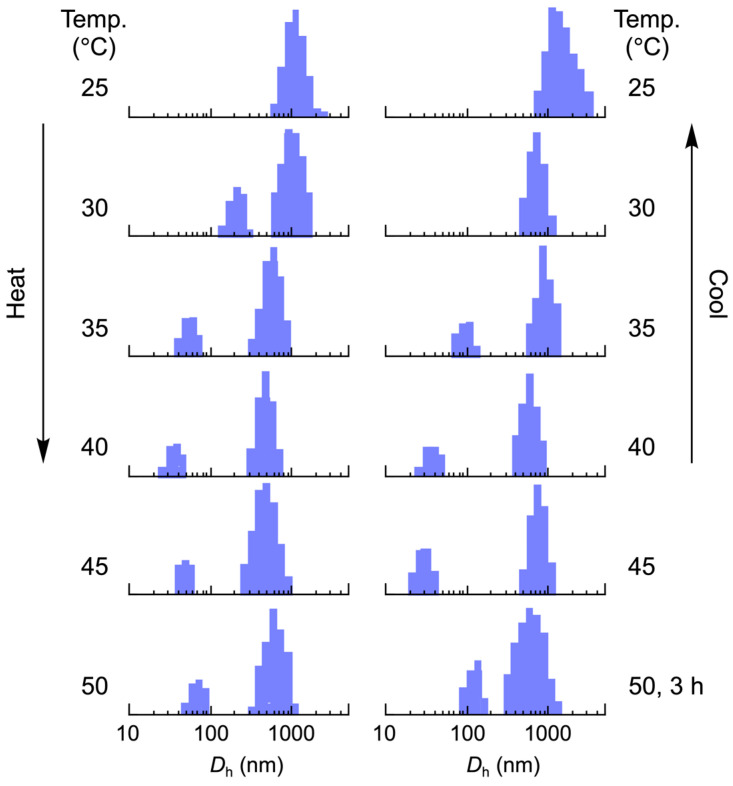
Scattering intensity distribution for the *D*_h_ of the vesicles at each temperature during heating and cooling. The vesicles are maintained at each temperature for 15 min, except at 50 °C for 3 h. CMA units = 30 mol%, [vesicle]_0_ = 0.267 g/L.

Scattering intensity distribution analysis supported this dissociation process. As shown in Figure 12, the vesicles partially dissociate on heating to 30 °C, producing small particles with a size of 192 nm. These particles further dissociated at 35 °C into much smaller particles with a 50 nm diameter, reducing the vesicle size. The distributions of the vesicles and particles continued to shift to smaller sizes upon heating to 40 °C; however, above this temperature, they shifted to larger sizes. Extending the heating period at 50 °C further shifted the distributions to larger sizes. Notably, the particle size increased from 64 nm to 111 nm after being maintained at this temperature for 3 h, suggesting the aggregation of the small particles. 

**Figure 13:**
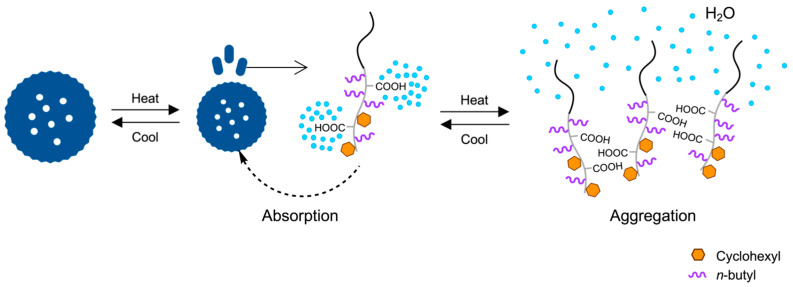
The dissociation of the copolymers, followed by their aggregation due to the loss of hydrated water molecules or absorption into the parent vesicle.

By annealing at high temperature, the P(BMA-*r*-CMA-*r*-MAA) blocks lose water molecules hydrated to the MAA units in the hydrophobic blocks, causing the blocks to aggregate with each other or be absorbed into their parent vesicles due to increased hydrophobicity (Figure 13). Upon cooling, the distributions returned to their original states, following the same course as the dissociation.

**Figure 14:**
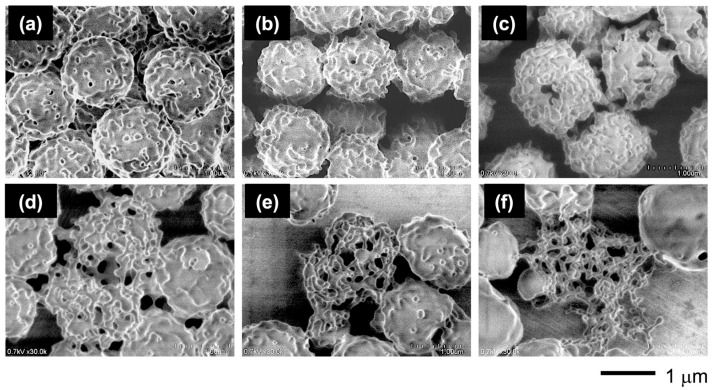
FE-SEM images of the vesicles with 30 mol% CMA units left at each temperature for 1 h: (**a**) 25 °C, (**b**) 30 °C, (**c**) 35 °C, (**d**) 40 °C, (**e**) 45 °C, and (**f**) 50 °C. [vesicle]_0_ = 2 g/L.

FE-SEM observations clarified the dissociation mechanism (Figure 14). The observations were made for the vesicles left at each temperature for 1 h. Upon heating, the rough surface enhanced the microphase separation to form holes in the shell, followed by the dissociation into tubule networks composed of triskelion-like segments. These segments were isolated from the networks by heating at 50 °C, producing much larger vesicles with smooth surfaces and no holes due to shell reorganization through segment fusion.

**Figure 15:**
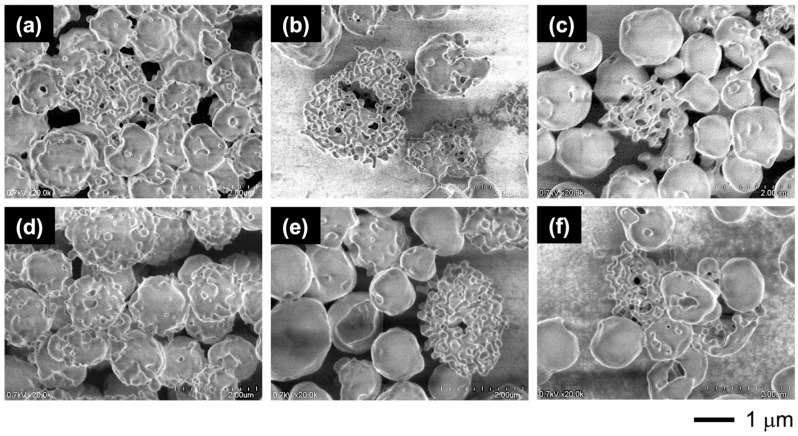
FE-SEM images of vesicles heated to 50 °C at different concentrations and annealing times: (**a**) 4 g/L, 1.5 h; (**b**) 2 g/L, 1.5 h; (**c**) 1 g/L, 1.5 h; (**d**) 4 g/L, 2 h; (**e**) 2 g/L, 2 h; and (**f**) 1 g/L, 2 h. CMA units = 30 mol%.

Extending the annealing at 50 °C with different vesicle concentrations confirmed this dissociation into triskelion segments and reorganization (Figure 15). Lowering the concentration promoted the dissociation but also increased the formation of smooth-surfaced vesicles through reorganization. Upon annealing the diluted vesicles at 50 °C, the rough-surfaced vesicles transformed into cages composed of triskelion-like segments, which were generated by phase separation on the surface. The triskelion-like segments further dissociated into spherical particles, releasing them from the tips. This dissociation mechanism suggests that the triskelion-like segment is a complex of the constituent copolymers.

**Figure 16:**
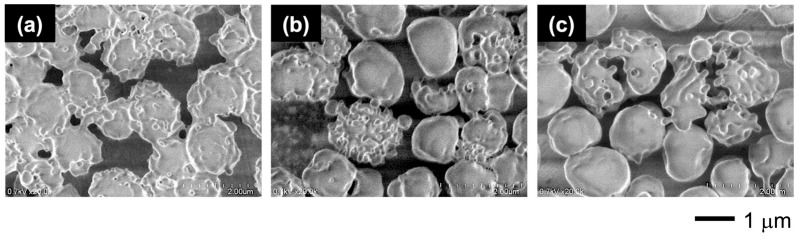
FE-SEM images of the vesicles left at 25 °C for 18.5 h at different concentrations: (**a**) 4 g/L, (**b**) 2 g/L, and (**c**) 1 g/L. CMA units = 30 mol%.

The dissociation–reorganization process was incompletely reversible as the disrupted vesicles did not revert to their original rough-surfaced state after cooling at room temperature for 18.5 h, remaining as smooth-surfaced vesicles and fused triskelion networks (Figure 16). It is likely that about 1 week is needed to restore them to their original state (Figure S2 in Supplementary Materials).

**Figure 17:**
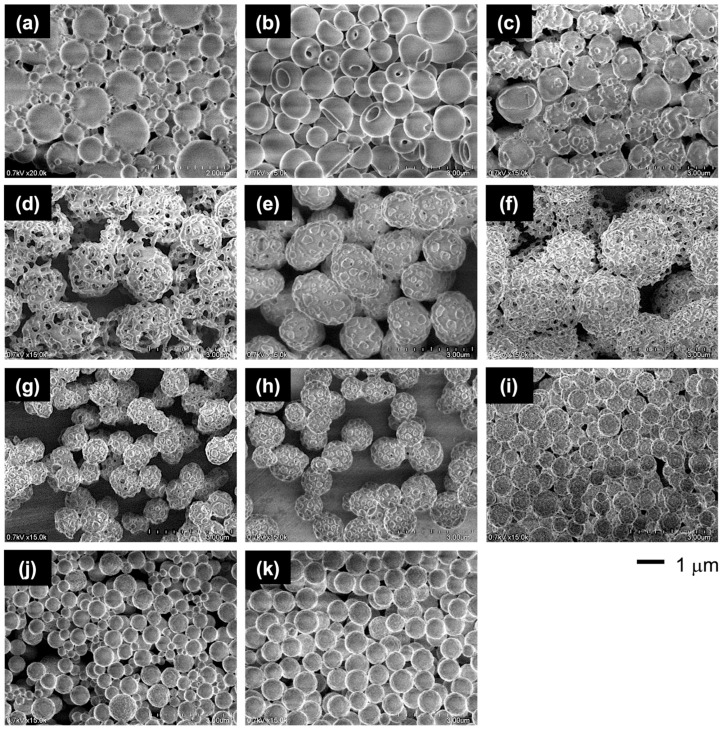
FE-SEM images of the vesicles heated at 50 °C for 1 h with different CMA unit ratios: (**a**) 0 mol%, (**b**) 10 mol%, (**c**) 20 mol%, (**d**) 30 mol%, (**e**) 40 mol%, (**f**) 50 mol%, (**g**) 60 mol%, (**h**) 70 mol%, (**i**) 80 mol%, (**j**) 90 mol%, and (**k**) 100 mol%.

The thermal stability of the vesicle skeleton was attributed to the hard CMA moieties. Figure 17 shows the morphologies of vesicles with different CMA ratios when heated at 50 °C. The vesicles with higher CMA ratios retained their morphologies, while those with lower CMA ratios transformed into cup-like vesicles or coarse-meshed cages. The thermal stability of the vesicles was also dependent on the chain length of the hydrophobic block. Even with a high CMA ratio, the vesicles containing the short hydrophobic block chains could not retain their morphologies and transformed into more contorted and fused vesicles at high temperatures (Figure S3 in Supplementary Materials). The hard CMA units also contributed to the stability of the vesicles against ionic strength (Figure S4 in Supplementary Materials). When placed in an aqueous NaCl solution, the vesicles with a low CMA ratio exhibited reduced shell flexibility, transforming into spherical or smooth-surfaced vesicles, whereas the vesicles with high CMA ratios retained their morphologies, maintaining the angular windows in their shells.

## 4. Discussion

The hard CMA units segregate from the hydrophobic block matrix, causing molecular deviation that enhances microphase separation, leading to the formation of holes in the vesicle shell. The hole formation, based on molecular deviation in the hydrophobic block, is supported by a previous study on perforated vesicle formation, where perforations in the vesicle shell resulted from partial interactions between different monomer units through an acid–base reaction in the hydrophobic phase [40]. In the present study, the deviation of the highly hydrophobic and hard CMA units promotes microphase separation. The CMA units at medium ratios, in the range of 40–80 mol%, formed holes in the vesicle shell. A ratio lower than 40 mol% of CMA units is insufficient for segregation, while a ratio higher than 70 mol% does not induce microphase separation. The CMA units at 40–70 mol% generate maximum microphase separation, forming large angular windows in the shell, which leads to the production of clathrin-like cage vesicles.

**Figure 18:**
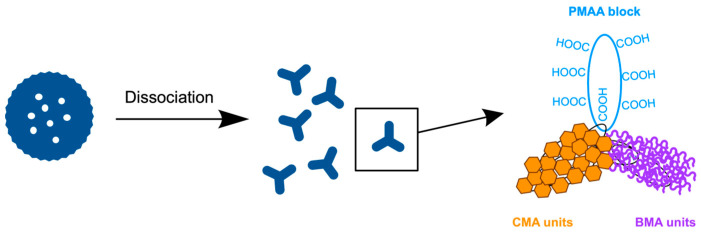
The dissociation of vesicles into triskelion-like segments undergoing intramolecular microphase separation.

The vesicles were extremely stable against changes in temperature and ion strength, making no changes in their morphology. However, the vesicles with a low CMA ratio less than 40 mol% dissociated upon heating into triskelion-structured segments. Clathrin triskelion is composed of three molecules from three different proteins, self-assembling into the triskelion structure. The reason the vesicles composed of a single type of linear copolymer dissociate into triskelion-like segments is that the isolated copolymer also undergoes intramolecular microphase separation (Figure 18). The copolymer contains four different components: the hydrophilic PMAA block, hydrophobically hard CMA units, hydrophobically soft BMA units, and hydrophilic MAA units in the random copolymer block. The MAA units interact with the PMAA block, accompanied by hydrated water molecules, while the hard CMA units segregate from the soft BMA unit matrix, leading to a triskelion-like structure. Increasing the temperature releases the water molecules from the MAA units and the PMAA block, strengthening the MAA-PMAA interaction and enhancing the segregation of the CMA units from the softened BMA units, which further advances triskelion formation.

The triskelion segments are attributed to their structural stability and flexibility. It has been reported that linear diblock copolymers containing a crystalline block, such as isotactic polypropylene-*block*-polystyrene (iPP-*b*-PS) with similar block lengths, self-assemble into an ordered bicontinuous double gyroid morphology—a continuous triskelion structure—due to microphase separation [49,50,51,52]. The formation of this morphology is attributed to the anchoring effect arising from the high crystal interhelix association of the iPP blocks. In the present study, the hard CMA units are expected to serve as anchors in the hydrophobic phase, promoting the triskelion structure.

Living organisms, including viruses, produce such stable and firm cages using proteins as amphiphiles rather than lipids or sugars to confine ingredients essential for survival or maintaining their life activities within their cages and to safely transport lipid-based transformable vesicles to target locations. Furthermore, considering that protein cages are composed of their own protein subunits with specific shapes in great variety [53], the findings of the present study indicate that synthetic polymers can also be designed to fabricate such shapes of subunits.

## 5. Conclusions

This study demonstrated that an amphiphilic diblock copolymer forms protein cage-like vesicles through polymerization-induced microphase separation. During the polymerization, the microphase separation was enhanced by the segregation of the hard units from the soft unit matrix, resulting in angular, large windows in the vesicle shell at the maximum occurrence of microphase separation at a medium ratio of the hard units. The vesicles with such windows are highly stable against external changes in temperature and ion strength. In contrast, the vesicles with a low content of the hard units dissociate upon heating into the triskelion-like segments. The formation of this triskelion structure was also attributed to intramolecularly generated microphase separation in the copolymer after its separation from the parent vesicle. Fabricating high-dimensional structures and morphologies of proteins using synthetic polymers holds promise for elucidating the significance of their formation.

## Figures and Tables

**Table 1:** Characterization of the diblock copolymers with varying CMA ratios synthesized by PISA via photo-NMP.

CMA Ratio ^1^ (mol%)	PMAA DP	Conversion (%)			Molar Ratio of Units			DP	*M*_n_	*M*_w_/*M*_n_
		BMA	CMA	MAA	BMA	CMA	MAA			
0	236	72	–	49	0.593	–	0.429	257	43,200	1.61
10	220	75	81	44	0.545	0.065	0.390	250	55,400	1.68
20	220	78	79	43	0.491	0.120	0.389	253	58,900	1.75
30	220	81	77	44	0.432	0.178	0.390	257	59,500	1.75
40	220	79	83	42	0.355	0.254	0.391	253	68,500	1.81
50	218	80	84	51	0.272	0.287	0.439	278	68,700	1.68
60	218	78	84	40	0.233	0.377	0.390	250	78,000	1.59
70	218	79	83	55	0.153	0.378	0.469	287	80,800	1.78
80	218	78	85	44	0.107	0.474	0.419	262	90,500	1.86
90	223	78	84	50	0.052	0.495	0.453	272	102,000	1.97
100	223	–	86	50	–	0.548	0.452	278	106,000	1.88

^1^ The molar ratio of CMA units to the total hydrophobic units (BMA and CMA) in the random copolymer block.

## Data Availability

The original contributions presented in the study are included in the article, further inquiries can be directed to the author.

## Supplementary Materials

The following supporting information can be downloaded at: https://www.mdpi.com/article/10.3390/ma18030727/s1, Materials; Table S1: Characterization of the diblock copolymers obtained at different polymerization times; Table S2: Monomers and their feed ratios used to evaluate the effect of the CMA unit on morphology; Figure S1: Thermal hysteresis of the vesicles; Figure S2: FE-SEM images of the restored vesicles; Figure S3: FE-SEM images of the vesicles with a short chain length in the hydrophobic block after heating at 50 °C for 1 h; Figure S4: F FE-SEM images of the vesicles left in NaCl aqueous solution.
