# Supplementary material for: Protein Cage-like Vesicles Fabricated via Polymerization-Induced Microphase Separation of Amphiphilic Diblock Copolymers
Source: Materials (Basel). 2025 Feb 6;18(3):727. doi: 10.3390/ma18030727 (PMC11820364; doi:10.3390/ma18030727)
Supplement: Supplementary file 1 [file materials-18-00727-s001.zip › materials-3424376-supplementary.pdf]

Supplementary Materials

Protein Cage-like Vesicles Fabricated via Polymerization-Induced Microphase Separation of Amphiphilic Diblock Copolymers

Eri Yoshida

Department of Applied Chemistry and Life Science, Toyohashi University of Technology

1-1 Hibarigaoka, Tempaku-cho, Toyohashi 441-8580, Japan

E-mail: yoshida.eri.gu@tut.jp

Content:

Experimental: Materials

Table S1: Characterization of the diblock copolymers obtained at different polymerization times.

Table S2: Monomers and their feed ratios used to evaluate the effect of the CMA unit on morphology.

Figure S1: Thermal hysteresis of the vesicles.

Figure S2: FE-SEM images of the restored vesicles.

Figure S3: FE-SEM images of the vesicles with a short chain length in the hydrophobic block after heating at 50 °C for 1 h.

Figure S4: FE-SEM images of the vesicles left in NaCl aqueous solution.

Materials:

MAA was purified by distillation under reduced pressure. BMA and CMA were purified using a conventional method: washed with 5% NaOH aqueous solution and water, dried with anhydrous $MgSO_4$, and distilled over calcium hydride under reduced pressure. The purified monomers were degassed with argon for 15 min with stirring just before use. V-61 and ^{t}BuS purchased from Wako Pure Chemical Industries and Sigma-Aldrich, respectively, were used as received. MeOH was purified by refluxing over magnesium with a small amount of iodine for several hours, followed by distillation. Distilled water was purchased from Wako Pure Chemical Industries and further purified by distillation. Nitrogen with a purity of over 99.9995 vol% and argon with a purity of over 99.999 vol% were purchased from Taiyo Nippon Sanso Corporation.

Tables:

Table S1. Characterization of the diblock copolymers obtained at different polymerization times.

Time (h)	PMAA DP	Conversion (%)			Molar ratio of units [1]			DP	M_n	M_w/M_n
		BMA	CMA	MAA	BMA	CMA	MAA			
2	215	35	39	8	0.290	0.492	0.218	90	48,600	1.83
3	215	48	60	29	0.208	0.388	0.404	174	56,400	1.74
5.5	215	75	80	38	0.235	0.381	0.384	238	71,500	1.74
8	218	78	84	40	0.233	0.377	0.390	250	78,000	1.59

[1] Molar ratio of units in the hydrophobic block.

Table S2. Monomers and their feed ratios used to evaluate the effect of the CMA unit on morphology.

CMA ratio [1] (mol%)	PMAA-MTEMPO		BMA (mmol)	CMA (mmol)	MAA (mmol)	Feed ratio		
	Conversion (%)	DP				BMA	CMA	MAA
0	76	236	3.961	–	4.325	0.478	–	0.522
10	81	220	3.521	0.3900	4.325	0.428	0.047	0.525
20	81	220	3.081	0.7449	4.442	0.373	0.092	0.535
30	81	220	2.641	1.146	4.442	0.321	0.139	0.540
40	81	220	2.200	1.490	4.560	0.267	0.180	0.553
50	79	218	1.823	1.834	4.643	0.220	0.221	0.559
60	79	218	1.446	2.177	4.643	0.175	0.263	0.562
70	79	218	1.069	2.521	4.761	0.128	0.302	0.570
80	79	218	0.6916	2.808	4.761	0.0837	0.3403	0.576
90	77	223	0.3458	3.094	4.754	0.0306	0.3894	0.580
100	77	223	–	3.438	4.872	–	0.414	0.586

[1] Molar ratio of CMA units to the total hydrophobic units (BMA and CMA) in the random copolymer block.

Figures:

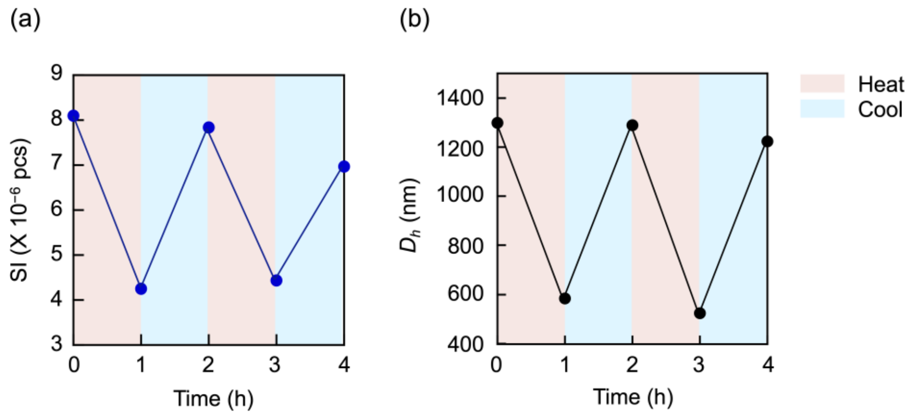

Figure S1. Thermal hysteresis of the vesicles: Variations in (a) scattering intensity (*SI*) and (b) hydrodynamic size (D_h) of the vesicles. CMA units = 30 mol%, $[\text{vesicle}]_0$ = 0.267 g/L.

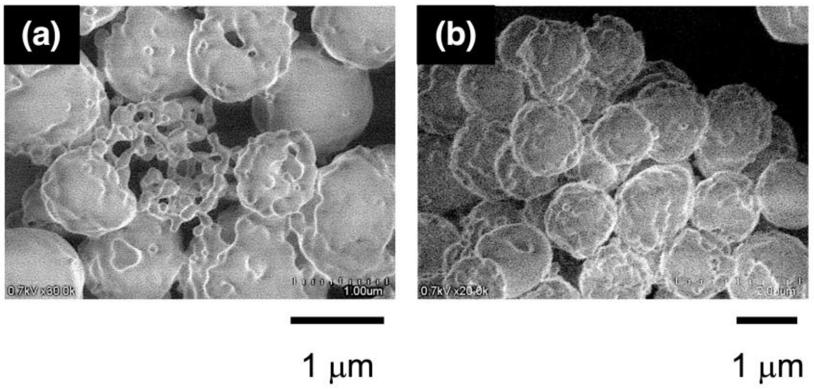

Figure S2. FE-SEM images of the restored vesicles: (a) Vesicles heated at 40 °C for 8 h, and (b) vesicles cooled at 25 °C for 1 week. CMA units = 30 mol%.

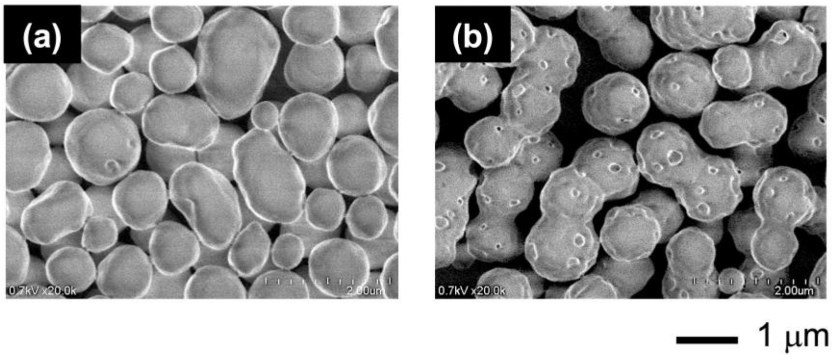

Figure S3. FE-SEM images of the vesicles with a short chain length in the hydrophobic block after heating at 50 °C for 1 h. Degree of polymerization (DP) of the hydrophobic block: (a) 90 for 2-h polymerization, and (b) 174 for 3-h polymerization.

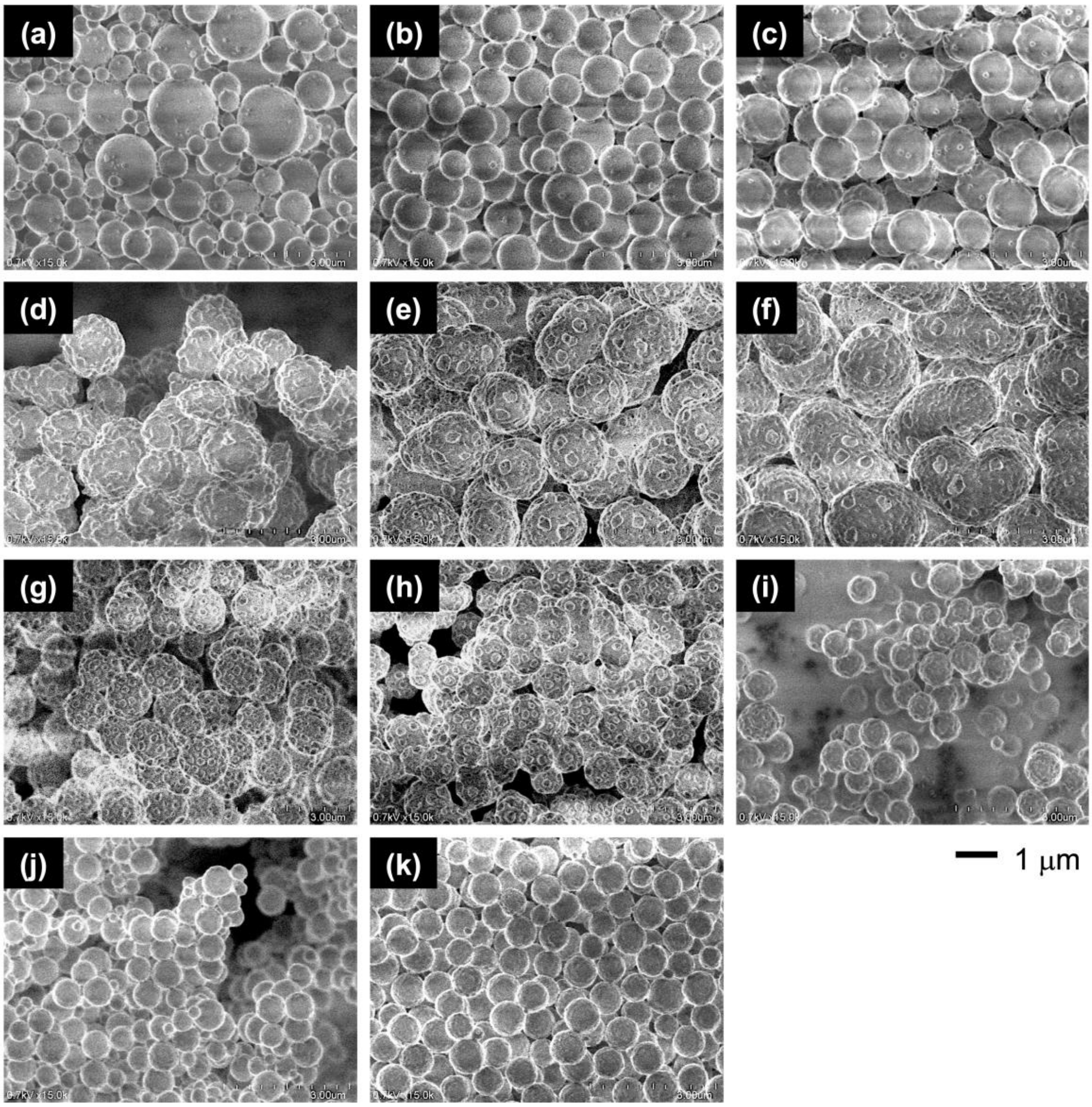

Figure S4. FE-SEM images of the vesicles left in NaCl aqueous solution. CMA unit ratio: (a) 0 mol%, (b) 10 mol%, (c) 20 mol%, (d) 30 mol%, (e) 40 mol%, (f) 50 mol%, (g) 60 mol%, (h) 70 mol%, (i) 80 mol%, (j) 90 mol%, and (k) 100 mol%. Conditions: $[NaCl]_0$ = 0.1 M, $[vesicle]_0$ = 7 g/L, 25 °C, 2 weeks.
